# A comprehensive proteomic analysis uncovers novel molecular subtypes of gastric signet ring cell carcinoma: Identification of potential prognostic biomarkers and therapeutic targets

**DOI:** 10.1016/j.gendis.2025.101717

**Published:** 2025-06-14

**Authors:** Zhiyuan Jin, Li Yuan, Yubo Ma, Zu Ye, Zhao Zhang, Yi Wang, Can Hu, Jinyun Dong, Xinuo Zhang, Zhiyuan Xu, Yian Du, Xiaoqing Guan, Guangzhao Pan, Sichao Tian, Juan Li, Ruiwen Zhang, Jiang-Jiang Qin, Xiangdong Cheng

**Affiliations:** a School of Life Sciences, Tianjin University, Tianjin 300000, China; b Zhejiang Cancer Hospital, Hangzhou Institute of Medicine, Chinese Academy of Sciences, Hangzhou, Zhejiang 310022, China; c Zhejiang Provincial Research Center for Upper Gastrointestinal Tract Cancer, Zhejiang Cancer Hospital, Hangzhou, Zhejiang 310022, China; d Key Laboratory of Prevention, Diagnosis and Therapy of Upper Gastrointestinal Cancer of Zhejiang Province, Hangzhou, Zhejiang 310022, China; e MOE Key Laboratory of Metabolism and Molecular Medicine, Department of Biochemistry and Molecular Biology, School of Basic Medical Sciences, Fudan University, Shanghai 200032, China; f Institute of Medicinal Plant Development, Chinese Academy of Medical Sciences and Peking Union Medical College, Beijing 100193, China; g Department of Pharmacological and Pharmaceutical Sciences, College of Pharmacy, University of Houston, Houston, TX 77204, USA

**Keywords:** Drug target, Gastric signet ring cell carcinoma, Molecular subtypes, Prognostic markers, Proteomics

## Abstract

Gastric signet ring cell carcinoma (GSRCC) is a distinct subtype of gastric cancer with unique epidemiological and pathogenic characteristics. However, its prognostic features and molecular landscape remain poorly understood, limiting the development of targeted therapies. In this study, we analyzed clinical data from over 10,000 patients with gastric cancer treated at Zhejiang Cancer Hospital between January 2010 and December 2019. A comprehensive proteomic analysis was conducted on 112 GSRCC patients with a signet ring cell content exceeding 70%, identifying 7322 proteins. This study established a tissue-specific peptide spectral library, representing the most extensive proteomic atlas of GSRCC to date. We identified four novel proteomic subtypes: metabolism, microenvironment dysregulation, migration, and proliferation. Furthermore, PRDX2 and DDX27 emerged as potential prognostic biomarkers, which were further validated in an independent cohort of 75 patients. Molecular profiling of 79 cases that lacked expression of established gastric cancer treatment targets and biomarkers revealed significant tumor heterogeneity. Unsupervised clustering identified three distinct proteomic clusters, with cluster 2 exhibiting the poorest prognosis. Additionally, we identified four potential drug targets, including PFAS, EIF2S3, EIF6, and NFKB2. Molecular docking analysis suggested that neratinib, a clinically approved drug, could serve as a promising therapeutic agent for GSRCC, offering new avenues for clinical intervention.

## Introduction

Gastric cancer (GC) is one of the most prevalent malignancies of the gastrointestinal tract, characterized by both a high incidence and mortality rate.^1^^,^^2^ Despite recent advancements in clinical treatment, including multi-drug combination regimens incorporating chemotherapy, targeted therapy, and immune checkpoint inhibitors,^3^^,^^4^ only certain subsets of patients with advanced GC have shown improved survival outcomes.^5^^,^^6^ For the majority of patients with advanced or recurrent GC, the prognosis remains poor.^2^^,^^7^ Notably, the incidence of gastric signet ring cell carcinoma (GSRCC) has been steadily increasing.^8^ Compared with other GC subtypes, GSRCC exhibits distinct biological characteristics, including immunosuppression, aggressive malignant progression, and multi-drug resistance.^9^^,^^10^ Histologically, GSRCC is defined by the presence of more than 50% dispersed cancer cells containing intracytoplasmic mucin. While previous studies have classified GSRCC as a diffuse-type GC, a comprehensive investigation into its molecular characteristics remains lacking. The intra-tumoral heterogeneity and low prevalence of signet ring cells in most tumor samples further complicate its characterization.^11^^,^^12^ Consequently, understanding the pathological and molecular features of GSRCC has become a critical focus in GC research.

Traditional GC classification methods have proven insufficient for guiding precise therapeutic decisions. Molecular classification is essential for personalized treatment.^13^, ^14^, ^15^ Based on clinical and molecular characteristics, GC is categorized into four subtypes: Epstein–Barr virus (EBV) positive, microsatellite instability, genomically stable, and chromosomal instability.^10^ While targeted and immunotherapeutic options exist for microsatellite instability, human epidermal growth factor receptor 2 (HER2)-positive, and EBV-positive GC,^16^ a significant subset of GSRCC patients is HER2-negative, EBV-negative, and proficient in mismatch repair (pMMR). These patients, identified in this study as Lack of Medical Treatment for Gastric Signet Ring Cell Carcinoma (LMT-GSRCC), currently lack effective treatment options, highlighting the urgent need for novel targeted therapies. The high tumor heterogeneity and limited therapeutic targets in LMT-GSRCC contribute to a median survival of only 15.9–20.8 months.^17^^,^^18^

Given the increasing incidence of GSRCC, a comprehensive molecular and proteomic investigation is urgently needed. Proteomics, an emerging tool in precision oncology, offers a holistic approach to analyzing GSRCC at the molecular level. However, the proteomic landscape of GSRCC remains poorly defined. To bridge the gap between basic research and clinical application, this study aims to characterize the proteomic features and molecular mechanisms of GSRCC, which holds significant implications for clinical practice. Using pressure cycling technology (PCT) coupled with data-independent acquisition mass spectrometry (DIA-MS), we conducted a comprehensive proteomic analysis on formalin-fixed paraffin-embedded (FFPE) GSRCC specimens, integrating clinical outcome data.^19^, ^20^, ^21^, ^22^ This approach enables the molecular characterization of GSRCC's complex heterogeneity, providing deeper insights into its phenotypic and biological underpinnings. Our study focuses on constructing proteomic maps and identifying molecular subtypes of GSRCC, providing valuable biomarkers for its precision management, while also uncovering potential therapeutic targets for LMT-GSRCC. By providing valuable insights for clinical stratification and targeted treatment strategies, this study contributes to advancing precision medicine in GC.

## Materials and methods

### Clinical cohort and specimen

This retrospective study focused on patients diagnosed with GC at Zhejiang Cancer Hospital between January 2010 and December 2019. The inclusion criteria were as follows: i) histologically confirmed adenocarcinoma, partial GSRCC (PGSRCC), or GSRCC, where GSRCC was defined by ≥ 50% signet ring cell content, PGSRCC by 1%–50% signet ring cells, and adenocarcinoma by the absence of signet ring cells; ii) availability of complete medical records; iii) survival follow-up data. Exclusion criteria included: i) GC patients diagnosed with other malignancies; ii) the presence of additional pathological components (*e.g.*, neuroendocrine carcinoma, squamous cell carcinoma).

A total of 11,899 GC patients were screened over the decade based on these criteria. Ultimately, 5422 GC patients were included in the study, comprising 3816 adenocarcinoma cases, 1330 PGSRCC cases, and 276 GSRCC cases. Among the 276 GSRCC cases, 112 patients with ≥70% signet ring cell content and no prior chemotherapy or radiotherapy were selected for proteomic analysis. Tumor tissues and paired normal adjacent tissues (NATs) were collected from these patients during surgical resection and preserved using the FFPE method. For independent validation, an additional cohort of 75 GSRCC patients (also with ≥70% signet ring cell content and no prior chemotherapy or radiotherapy) was collected under the same rigorous criteria at Zhejiang Cancer Hospital. The independent cohort was used to validate prognostic markers via immunoblotting, ensuring a comparable patient population for robust validation. This study is registered at *ClinicalTrials.gov* (NCT05985577).

### Clinical information and immunohistochemistry

Comprehensive clinical data were collected, including age, gender, family history, smoking and drinking statuses, surgery date, Lauren type, differentiation grade, tumor size, TNM staging, blood tumor markers, EBV status, survival status, and survival time, along with the expression of HER2, mutL homolog 1 (MLH1), postmeiotic segregation increased 2 (PMS2), mutS homolog 2 (MSH2), and mutS homolog 6 (MSH6).^23^^,^^24^ The following monoclonal antibodies were used for immunohistochemistry: MLH1 (Clone ES05, DAKO), PMS2 (Clone EP51, DAKO), MSH2 (Clone FE11, DAKO), and MSH6 (Clone EP49, DAKO). Loss of expression for any one of these proteins denotes deficient mismatch repair function (dMMR), while expression of all four indicates pMMR.

### Protein extraction and trypsin digestion assisted by PCT

Tissue samples were dewaxed, rehydrated, acidified, and rinsed with buffer before being processed in a PCT tube containing lysis and hydrolysis buffers. Enzymatic cleavage, reduction, and hydroxylation were performed under pressure cycling conditions, followed by digestion with trypsin and Lys-C. The resulting peptides were desalted using a C18 column, concentrated, and stored at −80 °C for further analysis.^25^

### Peptide separation via alkaline reverse-phase chromatography

To enhance peptide coverage in the library, high-pH reverse-phase chromatography was utilized for initial peptide separation. A pooled sample was created by combining 1 μL from each of the 224 individual samples. After quantification, 100 μg of the sample mixture was subjected to rotary evaporation until completely dry. The dried sample was then resuspended in approximately 105 μL of mobile phase A. Using the Dionex Ultimate 3000 system with an X Bridge peptide BEH C18 column (4.6 mm × 250 mm, 5 μm, 1/pkg), separation was carried out at a column temperature of 45 °C. The mobile phase consisted of buffer A (ddH_2_O with 0.6% ammonia, pH = 10) and buffer B (98% acetonitrile with 0.6% ammonia, pH = 10). A gradient elution from 3% to 35% buffer B was performed over 70 min at a flow rate of 1.0 mL/min, with fraction collection initiated at 10 min. 60 fractions (Fr) were collected at one per minute, which were then combined in an alternating manner: Fr.1 with Fr.31, Fr.2 with Fr.32, continuing until Fr.30 with Fr.60, yielding 30 combined fractions. The combined fractions were subjected to spin-drying using a centrifugal concentrator (6 mbar, 45 °C, 3 h). After drying, fractions were reconstituted in 20 μL of MS buffer A and centrifuged at 12,000 *g* at 4 °C for 15 min. Finally, 18.5 μL of the supernatant was carefully transferred into a plastic screw-cap sample vial for subsequent MS analysis.

### Data-dependent acquisition (DDA)-DIA library construction

The “DIA-DIA-Umpire_SpecLib_Quant” workflow was selected for library construction, integrating both DDA and DIA data. FragPipe (version 17.1) was utilized to generate project-specific libraries.^21^^,^^26^ The process involved several key steps: i) select the “DIA-DIA-Umpire_SpecLib_Quant” workflow and use DDA-DIA data to construct the library; ii) set ‘Max Missed Scans’ to 1, enable ‘Remove Background’, and apply the ‘Mass Defect Filter’ with a Mass Defect Offset of 0.1; iii) add the FASTA files obtained from https://www.uniprot.org/uniprot/, containing 20,377 reviewed protein sequences; iv) perform database searches using FragPipe to generate project-specific libraries.

The specific steps for database search were as follows: i) use FragPipe; ii) select the “DIA-DIA-Umpire SpecLib Quant” workflow; iii) add files, including the DIA file in mzML format, with the data type set to DIA; iv) configure Umpire settings: set Max Missed Scans to 1, select ‘Remove Background’ and ‘Mass Defect Filter’, set Mass Defect Offset to 0.1, and select ‘Q1’, ‘Q2’, and ‘Q3’; retain default settings for other parameters, while additional parameters were detailed in the developer documentation available at https://github.com/Nesvilab/FragPipe; v) include the required.fas file, following guidelines for database preparation found at https://github.com/Nesvilab/philosopher/wiki/How-to-Prepare-a-Protein-Database; vi) specify the output directory for results; vii) execute the process to produce ∗_Q1.mzML, ∗_Q2.mzML, and ∗_Q3.mzML files, simultaneously adding these files along with the DDA files, ensuring the data type was set to DDA and adhering to the workflow and parameter settings specified in steps 1 and 4.

### DIA data acquisition of samples

Proteomic data from 112 paired samples were acquired using DIA. The liquid chromatography gradient was maintained for 60 min, with buffer B concentrations ranging from 3% to 25% and a flow rate of 300 nL/min. The MS parameters for DIA included an *m*/*z* range of 390–1210 Th, a resolution of 60,000 full-width half-maximum (FWHM), an automatic gain control (AGC) target of 3E6, and a maximum ion injection time of 80 ms. For MS/MS scans, the resolution was set at 30,000 FWHM, with an AGC target of 1E6 and a maximum ion injection time of 55 ms. Twenty-four isolation windows were utilized, with all other settings maintained at default. For sample preparation, bicinchoninic acid concentration was adjusted to 0.2 μg/μL, and 20 μL was centrifuged at 12,000 rpm for 15 min. Of this, 18 μL was transferred to the injection vial. The injection volume was set at 2 μL, corresponding to a protein load of 0.4 μg, with a loading duration of 60 min. The order of typesetting was: Blank, pool, 1, 2, 3; blank, …, 12, 13, 14, 15.

### LC-MS/MS analysis

MS data analysis was conducted using FragPipe to generate sample-specific libraries, followed by DIA-NN (version 1.8) for querying the established GSRCC-specific spectral library. Fixed modifications included trypsin digestion, cysteine carbamidomethylation, and methionine excision at the N-terminus, with charge states ranging from 1 to 4. The fragment *m*/*z* range was established between 200 and 1800, and “Match between runs” was enabled. For quantification, “Any LC (high precision)” and “RT-dependent” served as cross-run normalization strategies. The proteomics output tables were filtered to a maximum of 1% Q-value at both the parent ion and global protein levels. Finally, multiple batches of MS data were processed using DIA-NN to create protein quantification matrices. The ggpubr package was utilized for data quality control analysis and statistical evaluation of matrix data, enabling a thorough quality assessment of the LC-MS/MS data.

### Statistical analysis

Proteins with a missing rate exceeding 35% were excluded from the matrix, and missing values were imputed using the K-nearest neighbor (KNN) package. The remaining identified proteins were used as input for differential expression analysis. A paired two-sided student's *t*-test was conducted, with the Benjamini-Hochberg correction applied to evaluate the statistical significance of differences between cancerous and paracancerous groups. A fold-change (Fc) threshold of 1.5 and an adjusted *p*-value of <0.05 were established. One-way ANOVA, followed by Benjamini-Hochberg correction, was used to compare protein expression across TNM stages (I, II, III, IV), with an adjusted *p*-value of <0.05 as the threshold for significance. Proteins from distinct clusters, identified through clustering analysis using mfuzz, were selected for further examination. A network diagram was constructed using the GeneMANIA plugin in Cytoscape (version 3.8.2). The principal component analysis (PCA) was performed on the T/N ratios of the top 100 differentially expressed proteins in each subtype, using the prcomp algorithm from the stats package in R. Visualization was achieved using the ggpubr package. Pathway enrichment analysis of clustered proteins was conducted using Ingenuity pathway analysis (IPA). The top 20 pathways, ranked by *p*-value, were selected for visual representation from eight identified clusters.

### Unsupervised subtyping using non-negative matrix factorization (NMF)

After excluding proteins with missing data in more than 35% of the 112 samples, the remaining proteins were imputed using the KNN algorithm from the DMwR package in R. Molecular subtyping was then performed using the NMF algorithm, with the tumor-to-adjacent tissue expression ratio as input data. The optimal classification was determined to consist of four subtypes. Following a similar procedure, proteins with missing data in more than 35% of the 79 samples were excluded, and the remaining proteins were imputed using the KNN algorithm. Subtyping was again conducted using the NMF algorithm, with the same input ratio, resulting in an optimal classification into three subtypes.

### Identification of subtype signature proteins

The relative expression levels of proteins in tumor tissues and NATs served as input data. Proteins exhibiting significant differences in expression across subtypes (F_C_ > 1.5, *p <* 0.05) were identified as subtype-specific signature proteins. Statistical significance was assessed using the Wilcoxon rank sum test (*p <* 0.05), and KEGG enrichment analysis was performed on the identified signature proteins using the R package clusterProfiler. The top 100 signature proteins, ranked by statistical significance, were selected for heatmap visualization.

### Statistical validation of proteomic subtypes' prognostic relevance

The prognostic significance of proteomic subtypes in GSRCC samples with extended follow-up was assessed using log-rank tests and Kaplan–Meier survival analysis. Additionally, the predictive accuracy of these subtypes was rigorously evaluated through univariable and multivariable Cox regression models. All statistical analyses were performed in R (version 3.2.1), with a significance threshold at 0.05.

Univariate and multivariate survival analyses were conducted using Cox regression models and stratified log-rank tests, with overall survival defined as the time from treatment initiation to death from any cause. Survival outcomes were visualized across different groups (subtype, cluster, and protein expression status) using Kaplan–Meier curves and forest plots. All tests were two-sided, with a significance threshold of 0.05. To control for type I errors, multiple comparisons were adjusted using the Benjamini-Hochberg false discovery rate (FDR) method. Kaplan–Meier survival curves for proteomic prognostic biomarkers were generated by categorizing cases according to median protein expression levels.

### Analysis of prognostic biomarkers for GSRCC

To identify prognostic biomarkers for GSRCC, the Cox proportional hazards model was applied to overall survival data. GSRCC patients were stratified into high- and low-expression groups based on the median protein expression level. Kaplan–Meier survival curves, supplemented by Log-rank tests, were used to illustrate survival differences. The criteria for selecting survival-related biomarkers included: tumor versus non-tumor with a *t*-test *p*-value < 0.0001 and Fc > 2, variance in tumor > 0.5, Log-rank *p*-value < 0.0001, and hazard ratio (HR) > 1.8 for up-regulated proteins or HR < 0.55 for down-regulated proteins.

### Analysis of potential drug targets for LMT-GSRCC

Potential drug targets for LMT-GSRCC were identified based on the following four criteria: i) Sub-type-specific expression: Candidate targets exhibited at least 1.5-fold higher expression in LMT-GSRCC subtypes compared with other subtypes and NATs (Wilcoxon signed-rank test, *p <* 0.05); ii) Candidate targets were limited to those approved by the FDA; iii) Univariate Cox analysis demonstrated that candidate targets were associated with an increased mortality risk (log-rank test, *p <* 0.05); and iv) Multivariate Cox analysis revealed that the combined candidate targets also demonstrated a significant association with higher mortality risk (log-rank test, *p <* 0.05).

### Virtual drug screening via molecular docking

The crystal structure of phosphoribosylformylglycinamidine synthase (PFAS) was retrieved from the UniProt protein database (PDB entry: O15067). Virtual screening was conducted using MOE (version 2019) software, with a small-molecule compound library provided by Tao Zhi Biological Inc. Protein and small-molecule libraries were prepared in MOE, and docking simulations were performed using the software's default experimental parameters. PyMOL was utilized for visualization of docking results.

### Assay of cell viability

The GSRCC cell lines were used to evaluate drug activity. Cells in the logarithmic growth phase were detached using trypsin-EDTA and seeded at 3 × 10^3^ cells per well in a 96-well plate (100 μL/well), followed by 12 h of incubation. DMSO was used as the negative control, while RPMI-1640 medium served as the blank control. Cells were exposed to gradient concentrations (0.625, 1.25, 2.5, 5, 10, 50, and 100 μM) for initial cytotoxicity screening of the following drugs: temsirolimus (CAS: 162635-04-3), bosutinib (CAS: 162635-04-3), infigratinib (CAS: 872511-34-7), pralsetinib (CAS: 2097132-94-8), and neratinib (CAS: 2097132-94-8). All drugs were sourced from Shanghai Taoshu Biotechnology Co., Ltd. After 48 h of incubation, 10% CCK8 reagent was added to each well, followed by 1–2 h of incubation. Absorbance at OD450 was measured using a microplate reader, and data were analyzed with GraphPad Prism (version 8.0). Each experiment was performed in triplicate (*n* = 3) for biological replicates.

### Colony formation assay

Cells were digested, resuspended via centrifugation, and counted before being seeded into 6-well plates. Experimental groups included biological replicates treated with different drug concentrations (1, 2, and 5 μM; *n* = 3), while DMSO served as the control. Cells were cultured until colonies consisting of at least 50 cells were observed through fixed staining. Two sets of 6-well plates were processed: the medium was discarded, and the cells were washed twice with phosphate-buffered saline solution and fixed with 1 mL of 4% paraformaldehyde per well. After a 20-min fixation, the fixative was removed, wells were washed with phosphate-buffered saline solution, and crystal violet staining solution was applied for 30 min. Subsequently, cells were rinsed with distilled water, air-dried, and photographed for statistical analysis.

### Cell migration assay

Exponentially growing healthy cells were trypsinized, counted, and seeded into migration assay plates at a density of 5 × 10^4^ cells per well. After cell adhesion, a uniform scratch was introduced across each well (*n* = 3). Wells were photographed at 0, 24, and 48 h to monitor cell migration into the scratched area.

### Cell invasion assay

For invasion assays, a layer of Matrigel was applied to the upper surface of the membrane. Cells were suspended in 200 μL of serum-free medium and seeded into the upper chamber of a Transwell system. In lower chamber contained 500 μL of complete medium supplemented with 20% fetal bovine serum to create a chemoattractant gradient. Experimental groups were treated with drug concentrations of 1, 2, and 5 μM, with biological replicates performed (*n* = 3), while DMSO served as the control. After 48 h of incubation, cells on the upper surface of the chamber membrane were removed using a cotton swab. The remaining invaded cells were washed with phosphate-buffered saline solution, stained with crystal violet, and photographed for analysis.

### Cell apoptosis assay

The apoptosis assay involved treating the experimental group with the compound of interest, while the control group remained untreated. After 48 h of incubation, both adherent and floating cells were collected and centrifuged at 1000 rpm for 5 min. The supernatant was discarded, and the cell pellet was resuspended in 200 μL of binding buffer. For flow cytometry analysis, each sample was incubated with 5 μL of FITC-conjugated annexin V and 5 μL of propidium iodide, followed by gentle vortexing and a 15-min incubation in the dark. Apoptotic cells were then quantified using a flow cytometer.

### Animal experiments

The study was approved by the ethics committee of Hangzhou Institute of Medicine, Chinese Academy of Sciences (approval number AP2024-09-0199), and all experiments were conducted in compliance with relevant guidelines and regulations. Female nude mice (aged 3–5 weeks) were housed in specific pathogen-free facilities at the Laboratory Animal Center of Hangzhou Institute of Medicine, Chinese Academy of Sciences. A precise amount of Neratinib powder was weighed and dissolved in a 15 mL centrifuge tube, with the solvent serving as the negative control. A cell suspension containing 8 × 10^5^ cells per mouse was injected subcutaneously. Upon confirmation of successful tumor inoculation, the mice were randomly assigned into three groups: the control group received intraperitoneal injections of the solvent (PEG400: normal saline: ethanol = 4: 2: 1), while the treatment groups received daily intraperitoneal injections neratinib at doses of 30 mg/kg and 60 mg/kg. Tumor size and body weight were recorded every two days. At the end of the study, major organs, including the heart, liver, spleen, lungs, and kidneys, were collected for hematoxylin-eosin staining to assess the potential toxicity. Processed tissue sections were examined under a microscope to identify any morphological or structural abnormalities. Additionally, a comprehensive biological safety evaluation included complete blood counts and liver function tests.

### Statistical analysis

Data analysis was conducted using GraphPad Prism software (version 8.0), with results expressed as mean ± standard error of the mean. Statistical comparisons between control and treatment groups were performed using *t*-tests, with *p*-values *<*0.05 considered statistically significant (∗*p <* 0.05, ∗∗*p <* 0.01, and ∗∗∗*p <* 0.001).

## Results

### GSRCC is a rare and intractable GC subtype with a poor prognosis

**Figure 1:**
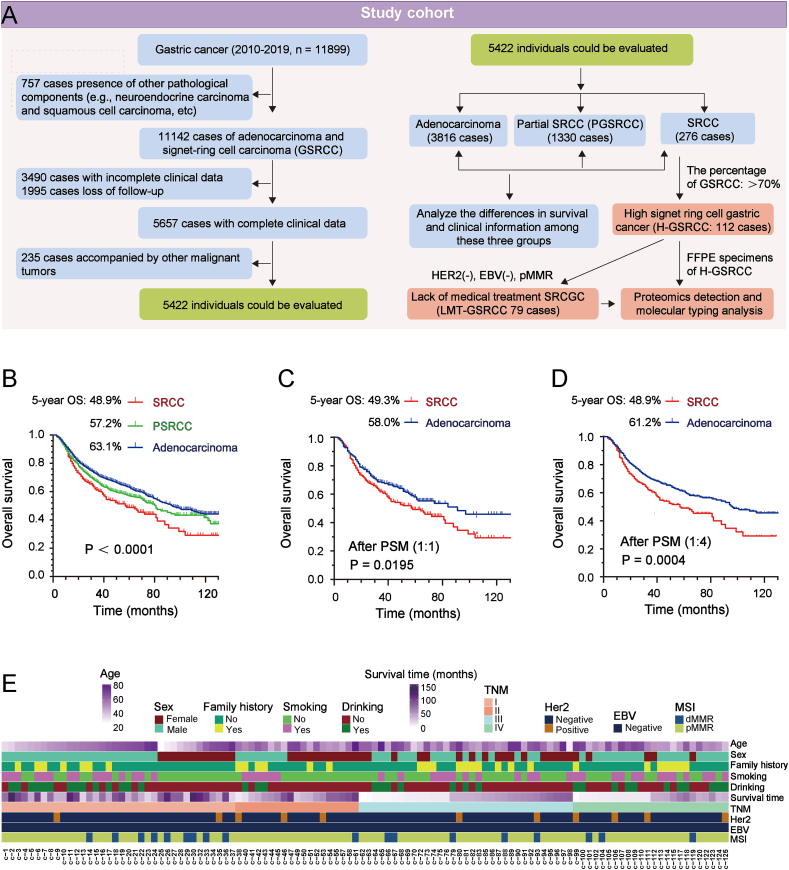
GSRCC exhibits a significantly lower survival rate compared with adenocarcinoma. **(A)** Study cohort enrollment and overview: A retrospective analysis included 5422 subjects, with 112 GSRCC patients selected for proteomic evaluation. **(B)** Kaplan–Meier survival analysis comparing GSRCC (*n* = 276), PGSRCC (*n* = 1330), and adenocarcinoma (*n* = 3816). **(C)** Kaplan–Meier survival analysis of GSRCC and adenocarcinoma groups following propensity score matching at a 1:1 ratio. **(D)** Kaplan–Meier survival analysis of GSRCC and adenocarcinoma groups following propensity score matching at a 1:4 ratio. **(E)** Clinical characteristics of the 112 GSRCC cases. GSRCC, gastric signet ring cell carcinoma; PGSRCC, partial GSRCC.

A preliminary screening of more than 10,000 GC patients diagnosed between 2010 and 2019 was conducted to identify eligible study participants (Fig. 1A). After applying stringent inclusion and exclusion criteria, a total of 5422 patients were enrolled in the study, including 3816 with adenocarcinoma, 1330 with PGSRCC, and 276 with GSRCC (Table S1). Clinical data, including age, sex, family history, and TNM stage, were collected for these patients. We observed that as the proportion of signet ring cells increased in adenocarcinoma, a trend emerged suggesting that patients with higher proportions of signet ring cells tended to be younger and predominantly female. In comparison to adenocarcinoma, patients with PGSRCC and GSRCC exhibited lower rates of smoking and alcohol consumption, likely reflecting gender differences (Table S2). Furthermore, GSRCC patients displayed distinctive features, including a higher incidence of vascular tumor thrombus, reduced nerve invasion, a greater proportion of patients in TNM stage I, fewer cases of recurrence and metastasis, and a higher number of tumors located in the distal stomach compared with adenocarcinoma (Table S2).

Significant differences in survival rates were observed among the three groups: adenocarcinoma, PGSRCC, and GSRCC. Adenocarcinoma exhibited the most favorable prognosis, with a 5-year survival rate of 63.1%. PGSRCC followed, demonstrating a 5-year survival rate of 57.2%. However, GSRCC showed the poorest prognosis, with a 5-year survival rate of only 48.9% (Fig. 1B–D and Table S3, 4). Therefore, GSRCC was identified as a GC subtype associated with a notably poor prognosis, warranting increased attention. Among the 276 GSRCC cases screened, 112 patients exhibited a signet ring cell content greater than 70%. Proteomics was conducted to analyze the molecular characteristics of GSRCC (Fig. 1E and Table S5). Additionally, testing for HER2, EBV, and DNA mismatch repair genes classified 79 of the 112 patients as LMT-GSRC, based on their HER2-negative, EBV-negative, and pMMR statuses (Table S6).

### Proteomic characteristics of GSRCC tumors

Tumor tissues and corresponding NATs from 112 GSRCC patients, each with a signet ring cell content exceeding 70%, were selected for in-depth proteomic analysis. PCT combined with DIA-MS enabled robust and reproducible proteomic profiling of biopsy-level FFPE tissues (Fig. S1A and Table S7), yielding high Pearson correlation values ranging from 0.928 to 0.981 across samples (Fig. S1B). Density distribution analysis of the raw data confirmed the absence of significant outliers among these 112 paired samples (Fig. S1C). The DIA-PCT assay achieved high protein quantification coverage, detecting over 80% of proteins with at least two peptides, with 70% consistently identified across all samples (Fig. S1D).

**Figure 2:**
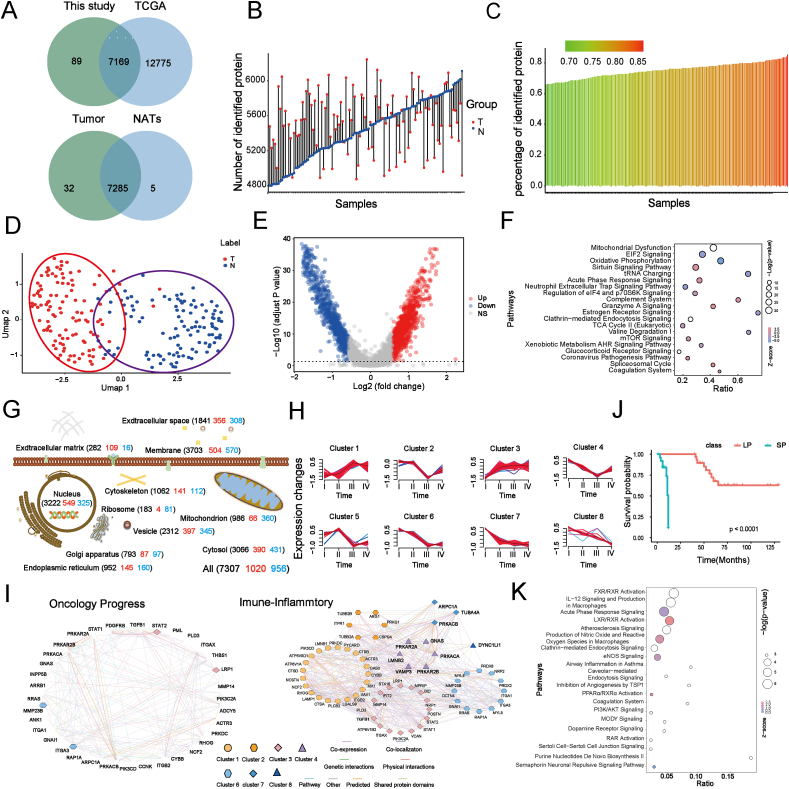
Integrating clinical features in proteomic stratification analysis of GSRCC. **(A)** Comparison of TCGA data with protein levels from this study, identifying overlapping proteins in tumors and NATs. **(B)** Number of proteins identified in each tumor and NATs pair, with red representing tumor samples and blue indicating NATs. **(C)** Proportions of proteins identified in each sample relative to the total detected proteins. Color coding indicates sample proportions: a shift toward blue represents a lower proportion, while a shift toward yellow signifies a higher proportion. **(D)** The volcano plot illustrating differentially expressed proteins, where the horizontal axis represents Log_2_(Fc) and the vertical axis represents −Log_10_ (adjusted *p* value). Up-regulated proteins were marked in red, down-regulated proteins in green, and non-significant proteins in gray. **(E)** Uniform manifold approximation and projection analysis of 112 paired FFPE GSRCC samples, using differentially expressed proteins to distinguish tumor samples (red) and NATs (blue). **(F)** Ingenuity pathway analysis (IPA) identified enriched pathways associated with differentially expressed proteins between tumors and NATs. **(G)** Subcellular distribution of GSRCC proteins, annotated using Gene Ontology. All proteins are represented in black, up-regulated proteins in red, and down-regulated proteins in blue. **(H)** Unsupervised clustering of proteome dynamics revealed six distinct protein expression patterns associated with GSRCC progression. Each line represents relative protein abundance, color-coded by cluster membership. **(I)** Protein–protein interaction (PPI) analysis of key proteins across different clusters based on unsupervised clustering. **(J)** Kaplan–Meier survival analysis comparing patients with long survival (LP group, *n* = 31, survival >12 months) and short survival (SP group, *n* = 26, survival <12 months), with *p*-values derived from the log-rank test. **(K)** IPA pathway enrichment analysis of differentially expressed proteins. GSRCC, gastric signet ring cell carcinoma; NATs, normal adjacent tissues; FFPE, formalin-fixed paraffin-embedded.

Further analysis of the 112 paired samples identified 7285 shared proteins between tumor samples and NATs, with 32 proteins exclusively present in tumors and 5 uniquely detected in NATs (Fig. 2A and Table S8). Proteomic profiling across all patient samples identified a total of 7322 proteins, all meeting quality control criteria (Fig. 2B, C). Differential expression analysis revealed 1985 (1034 up-regulated and 951 down-regulated) proteins with significant changes between tumors and NATs (paired two-sided student's *t*-test, BH-adjusted *p <* 0.05, Fc > 1.5) (Fig. 2D and Table S9). Uniform manifold approximation and projection analysis using differentially expressed proteins demonstrated clear separation between GSRCC tumors and NATs, while also revealing significant heterogeneity within tumor samples (Fig. 2E). No batch effects were detected.

IPA of differentially expressed proteins identified several key pathways associated with tumor immortality, including “mitochondrial dysfunction”, “oxidative phosphorylation”, “TCA cycle”, “mitochondrial fusion-mediated oxidative phosphorylation”, and “NADH/NAD^+^ metabolism”. Additionally, critical aging-related signaling pathways, such as “mTOR”, “AMPK”, “NF-κB”, and “sirtuins”, were significantly enriched. Notably, inflammatory response pathways, including “acute phase response signaling”, “neutrophil extracellular trap signaling pathway”, “coronavirus pathogenesis pathway”, “coagulation system”, and “xenobiotic metabolism AHR signaling pathway” were also highlighted. Furthermore, proteins related to genomic regulation and instability were enriched in the “spliceosome” and “tRNA charging” pathways (Fig. 2F).

To determine the subcellular localization of differentially expressed proteins, their organelle associations were analyzed. The results indicated that dysregulated proteins were predominantly expressed in the cell membrane, cytoplasm, and nucleus (Fig. 2G; Fig. S2A). Given their crucial role in GSRCC pathogenesis, membrane proteins warrant further investigation. Based on these findings, additional analyses were conducted to define specific protein expression patterns in GSRCC. Using Mfuzz clustering, we identified distinct protein clusters, which were visualized in a network diagram generated via the GeneMANIA plug-in in Cytoscape. Unsupervised clustering revealed eight biologically relevant protein expression patterns associated with GSRCC progression. Clusters 1 and 3 contained proteins that were consistently up-regulated with tumor progression, whereas clusters 2, 4, 5, 6, 7, and 8 comprised proteins with an overall down-regulation trend (Fig. 2H and Table S10). To elucidate the biological significance of these patterns, IPA was performed on proteins persistently up-regulated during tumor progression. This analysis revealed enrichment in immune response, proliferation, migration, and genomic regulatory instability pathways (Fig. S2B and Table S11). Protein–protein interaction network analysis further identified key regulators of these pathways, including core immune inflammatory proteins, inflammatory factors, and proteins linked to malignant tumor progression, underscoring their crucial roles in GSRCC development (Fig. 2I).

Expanding on these findings, we focused on advanced-stage GSRCC (stages III and IV) to investigate molecular differences underlying survival disparities (Table S12). Proteomic analysis compared long-survival patients (LP group, *n* = 31, survival > 12 months) and short-survival patients (SP group, *n* = 26, survival < 12 months) (Fig. 2J). Heatmap analysis of differentially expressed proteins revealed distinct molecular profiles between early-stage and advanced GSRCC, while also highlighting pronounced tumor heterogeneity (Fig. S2C). Using an adjusted *p*-value < 0.05 as the threshold, 522 differentially expressed proteins were identified. Pathway enrichment analysis indicated a strong association with mitochondrial dysfunction and oxidative phosphorylation (Fig. 2K). Notably, these pathways overlapped significantly with those implicated in GSRCC progression, suggesting their dual roles in both disease advancement and patient survival outcomes.

### Unsupervised proteomic analysis of GSRCC reveals clinically and biologically distinct clusters

**Figure 3:**
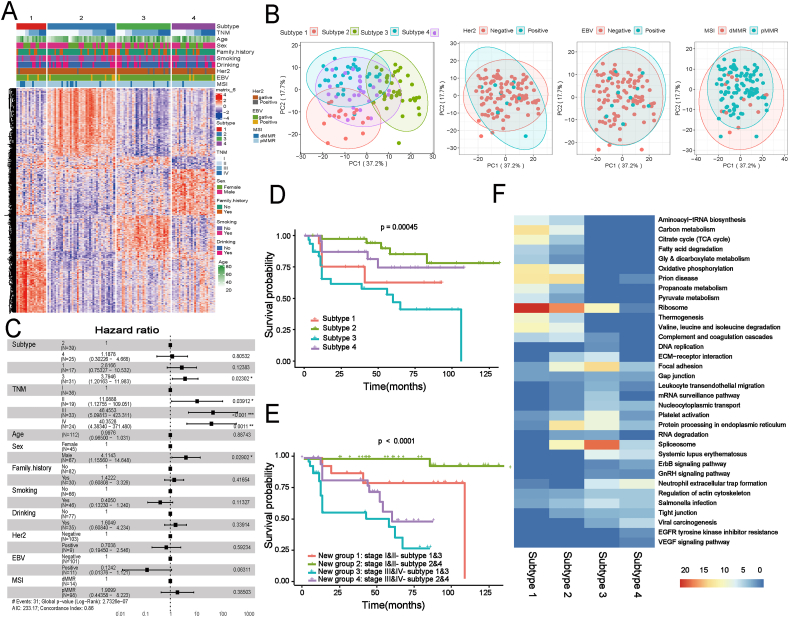
Proteomic analysis identifies distinct GSRCC subtypes with divergent clinical outcomes. **(A)** Unsupervised clustering of 112 GSRCC cases identified key proteins with significant differences between tumors and NATs, focusing on the top 100 proteins with a F_C_ > 1.5 and *p* value < 0.05. **(B)** Principal component analysis (PCA) visualizes the distribution of the identified subtypes, alongside HER2, EBV, and MSI status. **(C)** The Cox proportional hazards regression model was used to evaluate the impact of the newly identified subtypes and clinical factors on patient prognosis, calculating the hazard ratio (HR). **(D)** The associations between the four proteomic subtypes and overall survival in 112 GSRCC patients were assessed using the log-rank test, with Kaplan–Meier plots illustrating overall survival differences across subtypes. **(E)** Kaplan–Meier survival analysis stratified by TNM stage was further evaluated by overall survival variations among GSRCC subtypes. **(F)** Gene Set Enrichment Analysis (GSEA) identified significantly enriched biological pathways across GSRCC subtypes, with normalized scores highlighting key functional processes (adjusted *p* value < 0.05). GSRCC, gastric signet ring cell carcinoma; NATs, normal adjacent tissues; EBV, Epstein–Barr virus; HER2, human epidermal growth factor receptor 2; MSI, microsatellite instability.

We further explored the molecular subtypes of GSRCC based on the proteomics data. Focusing on protein levels, GSRCC were classified using unsupervised clustering, revealing four distinct subtypes based on the most variable proteins (Fig. 3A; Fig. S3A and Table S13). While these subtypes exhibited similarities in clinicopathological features such as age, smoking, drinking, and family history, significant differences were observed in TNM stage and sex distribution (Fig. 3C and Table S14–16). PCA of the top 100 differentially expressed proteins per subtype revealed that the first principal component accounted for 37.2% of the variance, while the second accounted for 17.7%. The PCA plot distinctly separated subtypes 1, 2, and 3, whereas subtype 4 displayed an intermediate gene expression pattern (Fig. 3B), which correlated with survival outcomes (Fig. 3D). Notably, traditional biomarkers such as Her2, EBV, and MSI failed to differentiate these subtypes in PCA visualizations (Fig. 3B), highlighting the advantages of this proteomics-based classification.

Kaplan–Meier survival analysis demonstrated significant differences among the subtypes, with overall survival varying considerably (*p* = 0.00045, log-rank test). Subtype 2 (*n* = 39) exhibited the most favorable prognosis, while subtype 3 (*n* = 31) had the worst overall survival (Fig. 3D). Importantly, stratifying patients by TNM stage confirmed that the proteomic subtypes remained significantly associated with survival outcomes, independent of tumor stage (log-rank test, *p <* 0.0001), underscoring the prognostic value of this classification system (Fig. 3E). Further analysis of the GSRCC proteomic landscape identified four distinct subtypes: metabolism (S-Mb), microenvironment dysregulation (S–Me), migration (S-M), and proliferation (S-PF). These subtypes exhibited significant differences in clinical prognosis. Notably, the migration (S-M) subtype, characterized by proteins involved in extracellular matrix remodeling and genomic instability signaling pathways, correlated with the poorest survival outcomes (Fig. 3F; Fig. S3B).

**Figure 4:**
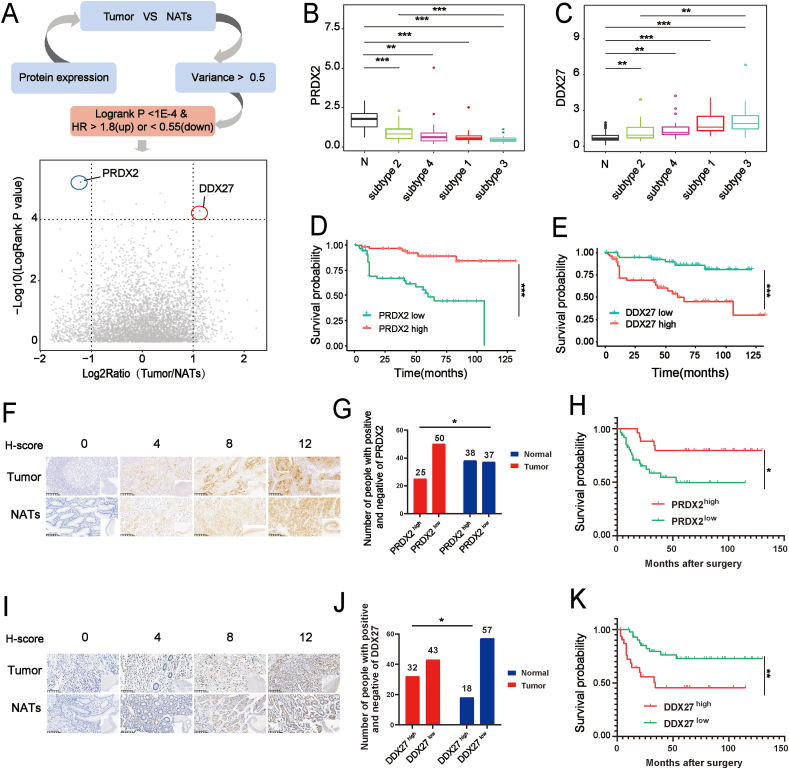
Identification and validation of prognostic proteomic biomarkers. **(A)** Workflow for selecting prognostic proteins, with candidate proteins represented as dots, annotated with their F_C_ and hazard ratio (HR). **(B, C)** PRDX2 and DDX27 abundance in tumor tissues versus NATs was assessed using *t*-tests, while differences across proteomic subgroups were analyzed via ANOVA. Box plots display median values and interquartile ranges. **(D, E)** Kaplan–Meier survival curves for overall survival based on PRDX2 and DDX27 proteomic abundance (*n* = 159; solid lines), with *p* values from log-rank tests. **(F)** Immunohistochemical staining of PRDX2 in tumors and NATs. **(G)** PRDX2 expression levels in a validation cohort of 75 cases with gastric signet ring cell carcinoma. **(H)** Kaplan–Meier survival curves for overall survival based on PRDX2 immunostaining scores in the validation cohort (*n* = 75). **(I)** Immunohistochemical staining of DDX27 in tumors and NATs. **(J)** DDX27 expression levels in the validation cohort. **(K)** Kaplan–Meier survival curves for overall survival based on DDX27 immunostaining scores in the validation cohort (*n* = 75). ∗*p <* 0.05, ∗∗*p <* 0.01, and ∗∗∗*p <* 0.001. PRDX2, peroxiredoxin 2; DDX27, DEAD-box helicase 27; NATs, normal adjacent tissues.

The OS data from 112 GSRCC patients were analyzed using the Cox proportional hazards model to identify potential prognostic biomarkers. Supervised analysis identified robust and representative prognostic proteins. After stringent filtering, one up-regulated protein, DEAD-box helicase 27 (DDX27; HR = 1.895; *p <* 0.001), and one down-regulated protein, peroxiredoxin 2 (PRDX2; HR = 0.088; *p* = 0.001), were selected (Fig. 4A and Table S17). Both proteins demonstrated significant differential expression across proteomic subgroups (Fig. 4B, C). Stratification of patients based on median protein expression levels revealed significant survival differences for PRDX2 and DDX27 (Fig. 4D, E). To validate these findings, an independent cohort of 75 GSRCC cases, each with a signet ring cell content exceeding 70%, was selected from a larger pool of 276 GSRCC cases (Fig. 1A and Table S18). Expression patterns and prognostic relevance of PRDX2 and DDX27 were further confirmed in this validation cohort. Consistent with the initial observations, PRDX2 was down-regulated in GSRCC and correlated with a favorable prognosis (Fig. 4F–H and Table S19), whereas DDX27 was up-regulated and associated with poor prognosis (Fig. 4I–K and Table S19). These findings underscore the significant prognostic value of PRDX2 and DDX27, highlighting their potential as clinical biomarkers for GSRCC treatment.

### Unsupervised proteomic analysis of LMT-GSRCC identifies clinically and biologically distinct clusters

**Figure 5:**
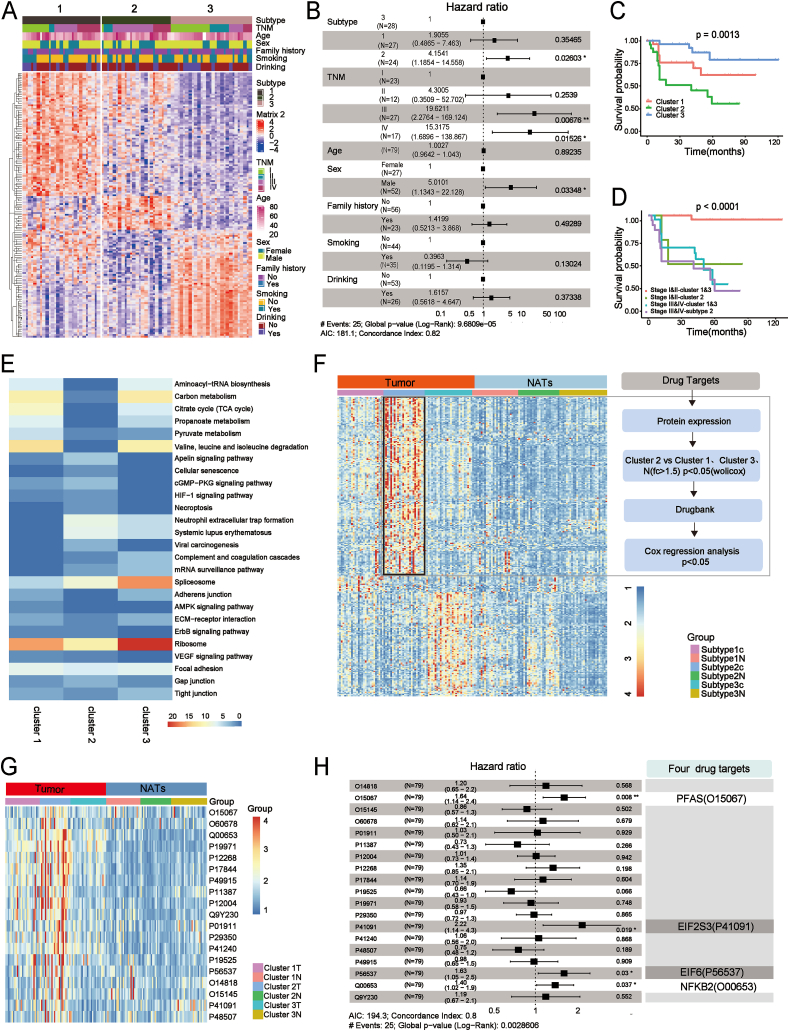
Proteomic signatures reveal biological heterogeneity in LMT-GSRCC. **(A)** Consensus clustering was performed on 79 cases, identifying proteins with the most distinct “Tumor tissues to NATs” ratio values within each cluster, focusing on the top 100 proteins with a ratio >1.5 and *p <* 0.05. **(B)** Clinical outcomes associated with proteomic clusters were analyzed using the Cox proportional hazards regression model to determine hazard ratios (HR). **(C)** Kaplan–Meier survival curves and log-rank tests assessed overall survival differences among the identified clusters in 79 LMT-GSRCC patients (*n* = 79). **(D)** Kaplan–Meier survival analysis further examined overall survival across LMT-GSRCC clusters after stratifying patients by TNM stage (*n* = 79). **(E)** Gene Set Enrichment Analysis (GSEA) identified significantly enriched pathways in each cluster, with adjusted *p* < 0.05. **(F)** A systematic screening approach identified potential drug targets specific to cluster 2 in LMT-GSRCC patients. **(G)** Key signature proteins within cluster 2 were identified. **(H)** Multivariate Cox regression analysis demonstrated that candidate drug targets significantly increased mortality risk (log-rank test, *p <* 0.05). GSRCC, gastric signet ring cell carcinoma; NATs, normal adjacent tissues; LMT-GSRCC, lack of medical treatment GSRCC.

A comprehensive analysis of tumor proteomics, including differential protein expression and underlying molecular mechanisms, may unveil promising therapeutic targets for GSRCC. In this study, we selected LMT-GSRCC cases (defined as HER2-negative, EBV-negative, and pMMR) from a cohort of 112 GSRCC patients through proteomic stratification and biological subtyping, offering insights for diagnosis and personalized treatment. Further clustering using an NMF algorithm aimed to establish correlations between proteomic clusters and clinical parameters (Table S20). Consensus clustering of 79 qualified cases identified three main clusters: LMT-Cluster-1 (*n* = 27), LMT-Cluster-2 (*n* = 24), and LMT-Cluster-3 (*n* = 28) (Fig. S4A and Table S21, 22). Proteomic clustering of LMT-GSRCC emerged as an independent prognostic factor, retaining significance even after adjustment for other clinicopathological variables in a multivariate Cox regression analysis (Fig. 5A, B and Table S23). PCA of the top 100 protein T/N ratios in the 79 cases revealed distinct expression patterns among clusters, with the first principal component explaining 46.4% of the variance, highlighting the effectiveness of this classification (Fig. S4B). Kaplan–Meier survival analysis demonstrated significant differences among clusters (*p* = 0.0013, log-rank test), with cluster 3 exhibiting the highest survival rate and cluster 2 the lowest (Fig. 5C). Furthermore, proteomic clustering remained a significant prognostic factor independent of tumor stage (log-rank test, *p <* 0.0001), reinforcing the predictive power of this proteomic approach (Fig. 5D). LMT-GSRCC cases were categorized into three distinct clusters based on proteomic profiles: the genetic instability cluster (C-Gi), the metabolism subgroup (C-Mb), and the migration-proliferation subgroup (C-Pf) (Fig. 5E; Fig. S4C). Each cluster was enriched in extracellular matrix remodeling and migration-related signaling pathways, reflecting the highly invasive and metastatic nature of GSRCC. Notably, the C-Pf cluster, characterized by elevated expression of proliferation markers such as PARP1, TOP2A, PCNA, and MKI-67, correlated with the poorest prognosis. Among the LMT-GSRCC clusters, particular attention was given to cluster 2 due to its distinct malignant behavior and limited therapeutic options. To identify potential drug targets, a rigorous biological screening process was conducted to pinpoint signature proteins specific to cluster 2, which may serve as therapeutic targets and offer new clinical treatment strategies (Fig. 5F, G and Tables S24, 25). Patients with elevated protein levels in LMT-GSRCC exhibited significantly reduced survival rates and higher mortality risk scores. Notably, four proteins, including PFAS, eukaryotic translation initiation factor 2 subunit gamma (EIF2S3), eukaryotic translation initiation factor 6 (EIF6), and nuclear factor kappa B subunit 2 (NFKB2), emerged as potential drug targets, with established FDA-approved treatments or ongoing clinical trials (Fig. 5H). High expression of these proteins was associated with poor prognosis, underscoring their relevance as promising therapeutic candidates.

### Molecular docking identifies potential therapeutic agents

**Figure 6:**
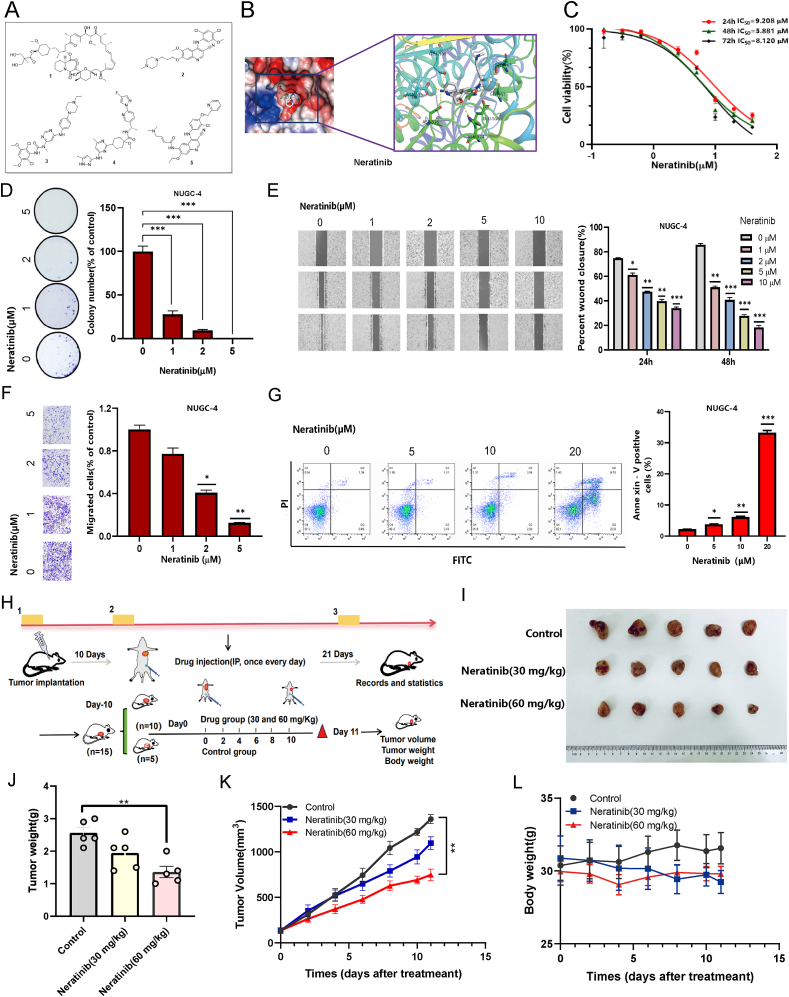
Molecular docking-based virtual screening of potential therapeutic agents for LMT-GSRCC. **(A)** Chemical structures of the five candidate drugs. **(B)** The docking model illustrating neratinib binding to PFAS protein domains. **(C)** Neratinib exhibited inhibitory effects on cell viability in the NUGC-4 cell line at varying concentrations over 24, 48, and 72 h. **(D)** Colony formation assays of NUGC-4 cells treated with neratinib at specified concentrations, with quantitative comparisons to the control group's colony formation rates (*n* = 3). **(E)** Migration distances were measured at 0, 24, and 48 h post-neratinib treatment. Quantitative analysis displays neratinib concentration on the *x*-axis and the relative migration distance percentage compared with the control group on the *y*-axis (*n* = 3). **(F)** Number of NUGC-4 cells that invaded the lower chamber. Quantitative analysis presents neratinib concentration on the *x*-axis and the proportion of migrating cells relative to the control group on the *y*-axis (*n* = 3). **(J)** Effect of neratinib on NUGC-4 apoptosis, with intervention concentrations on the *x*-axis and the total proportion of early and late apoptotic cells on the *y*-axis (*n* = 3). **(H)** Experimental workflow for *in vivo* anti-tumor efficacy assessment of neratinib. **(I)** Tumor size comparison between vehicle- and neratinib-treated groups on days 0 and 11. **(J)** Histogram of tumor weight comparing treatment and vehicle groups (*n* = 5). **(K)** Tumor volume curve, with time on the *x*-axis and tumor volume on the *y*-axis (*n* = 5). **(L)** Average mouse body weight plotted over time, with time on the *x*-axis and average weight on the *y*-axis (*n* = 5). ∗*p <* 0.05, ∗∗*p <* 0.01, and ∗∗∗*p <* 0.001. GSRCC, gastric signet ring cell carcinoma; LMT-GSRCC, lack of medical treatment GSRCC; PFAS, phosphoribosylformylglycinamidine synthetase.

To explore potential therapeutic options, molecular docking was conducted on candidate targets, including PFAS, EIF2S3, EIF6, and NFKB2, with PFAS emerging as the most significant target for further investigation (Fig. 5H and Table S26). Virtual screening identified five top-scoring drug candidates: temsirolimus, bosutinib, infigratinib, pralsetinib, and neratinib (Fig. 6A; Fig. S4E, F). Cytotoxicity assays demonstrated that both infigratinib and neratinib inhibited GSRCC cell growth in a concentration-dependent manner (Fig. S4D). Among these, neratinib exhibited the most potent inhibitory effect, followed by infigratinib. Despite limited investigation in clinically relevant GC models, preliminary findings suggest that neratinib holds promise as a novel therapeutic option for GSRCC.

To elucidate the molecular interaction between neratinib and PFAS, PyMOL-based structural analysis was performed, revealing a robust hydrogen bond network involving key amino acid residues such as asparagine (Fig. 6B). This interaction likely enhances neratinib's binding affinity to PFAS, correlating with its observed anti-tumor activity. The CCK8 assay further confirmed that neratinib inhibited NUGC-4 cell growth in a concentration-dependent manner (Fig. 6C). Moreover, neratinib significantly impaired colony formation in the NUGC-4 cell line, with no colonies detected microscopically at its IC_50_ concentration (Fig. 6D). Functional assays demonstrated neratinib's inhibitory effects on cell migration and invasion. In the wound-healing assay, the scratch closure rate of NUGC-4 cells decreased from approximately 80%–18% (Fig. 6E). Similarly, the transwell invasion assay revealed a substantial reduction in invasion rates upon treatment, with rates decreasing to 13.38% and 1.26% at 2 μM and 5 μM neratinib, respectively (Fig. 6F). Apoptosis analysis further indicated that at the highest tested concentration, neratinib induced apoptosis in 33.5% of treated cells (Fig. 6G). Collectively, these findings highlight neratinib's potential as an effective anti-tumor agent against GSRCC.

To further assess neratinib's therapeutic efficacy *in vivo*, a subcutaneous tumor model was established. Mice were treated with either vehicle control or neratinib (30 and 60 mg/kg) via intraperitoneal injection, and tumor growth was monitored by measuring tumor volume (Fig. 6H). While the control group exhibited continuous tumor progression, neratinib treatment resulted in significant tumor growth inhibition, evident from the first week through the study's conclusion (Fig. 6I). The high-dose neratinib group demonstrated statistically significant tumor suppression compared with the control group (∗*p <* 0.05) (Fig. 6J, K).

To evaluate potential toxicity, the body weight of mice was monitored throughout the study, revealing no significant differences between the control and neratinib-treated groups, suggesting a favorable safety profile at therapeutic doses (Fig. 6L). Further assessments of neratinib's safety profile included histopathological examinations, which showed no morphological or structural abnormalities in major organs. Additionally, complete blood counts and liver function tests revealed no significant alterations in key hematological parameters, including red blood cells, white blood cells, hemoglobin concentration, and platelets, or liver enzymes alanine aminotransferase and aspartate aminotransferase compared with the control group (Fig. S4G, H). While no statistically significant weight changes were observed (Fig. 6L), slight fluctuations suggest that potential cytotoxic effects should not be overlooked in future investigations.

Taken together, both *in vitro* and *in vivo* findings underscore neratinib's potential as a promising therapeutic agent for GSRCC. However, further studies are warranted to fully elucidate its long-term safety and efficacy.

## Discussion

Epidemiological data tracking indicates a notable annual increase in the incidence of GSRCC. However, systematic proteomic studies on this disease remain limited, constraining our understanding at the molecular level. Advanced MS-driven proteomics is crucial for identifying key protein biomarkers and potential drug targets, emphasizing the urgent need for comprehensive research to elucidate GSRCC pathogenesis and enhance therapeutic strategies. MS data acquisition has evolved significantly, with DDA and DIA being the primary methodologies in proteomics research.^26^, ^27^, ^28^ Over time, derivative techniques based on these core methods have been developed to enhance the analysis of complex datasets. DDA, an earlier technique, selectively identifies peptides with high peak intensity, ensuring clear and precise results. In contrast, DIA captures a broader spectrum of peptides within a predefined *m*/*z* range, leading to more comprehensive yet complex datasets that present challenges in downstream library searches.^29^ In our study, the complexities of DIA data were particularly evident. The broad, non-specific nature of the pan-human database occasionally resulted in inaccurate ion matching and protein misidentification, especially in tissue- and disease-specific studies. These findings underscore the necessity for tailored peptide libraries that can better accommodate tissue- and disease-specific variations.^26^ To address this issue, we constructed a GSRCC-specific spectral library for DIA analysis, generating an extensive proteomic profile of the disease. Analysis of 112 paired samples identified over 4800 proteins, which, after stringent quality control, yielded a comprehensive dataset comprising 7322 unique proteins, with a missing rate of 25.1%.

In this study, proteomic profiling of GSRCC revealed significant alterations in protein expression, including the emergence of novel proteins associated with disease progression. Integrating these findings with clinical data, we conducted an extensive bioinformatics analysis that identified GSRCC-specific cancer-related pathways. Notably, significant metabolic alterations were observed, particularly in amino acid and fatty acid metabolism, with elevated levels of these metabolites in NATs compared with tumor tissues. This metabolic imbalance, a previously underexplored hallmark of GSRCC, warrants further investigation. Prior research on GSRCC has primarily focused on histological differentiation and chemoresistance.^30^, ^31^, ^32^ A recent study by Shu et al highlighted the clinical significance of signet ring cell content and the role of claudin 18 (CLDN18)-Rho GTPase activating protein 26/6 (ARHGAP26/6) fusion in chemotherapy response.^33^ Similarly, Chen et al emphasized the role of C-X-C motif chemokine ligand 13 (CXCL13) in the tumor immune microenvironment and its contribution to adaptive immune unresponsiveness in GSRCC.^34^ While these studies have advanced our understanding of histological and immune aspects, recent research is beginning to uncover the molecular landscape of GSRCC. Our proteomic analysis across various disease stages suggests that factors such as “genomic regulatory instability”, “immunity”, “proliferation”, and “oncogenic signaling pathways” (*e.g.*, TNF-α, mTOR) may contribute to the poor prognosis associated with GSRCC. Instead, GSRCC has been identified as a significant negative prognostic factor in advanced GC following curative resection.^35^

To further dissect the heterogeneity of advanced GSRCC, we performed proteomic analyses on stage III and IV patients with diverse survival outcomes. This stratified approach, integrated with clinical data, provides valuable insights into both molecular and clinical characteristics of the disease. Leveraging an unsupervised consensus clustering algorithm, we identified four distinct proteomic subtypes of GSRCC, each exhibiting unique molecular signatures associated with clinical, pathological, and prognostic traits. Notably, subtypes with enhanced energy metabolism were linked to better prognosis, whereas those related to cell adhesion and proteome stability were indicative of poorer outcomes. Specifically, the S-III (S–Me) subtype, characterized by extracellular matrix dysregulation and genomic instability, exhibited the worst prognosis, consistent with previous findings associating these features with adverse clinical outcomes.^36^

Our findings further emphasize the importance of subtyping in precision oncology for GSRCC, identifying PRDX2 and DDX27 as potential prognostic markers. PRDX2 plays a dual role in tumorigenesis, acting as either a tumor suppressor or promoter depending on cellular context, with its expression influenced by etiology, cancer type, and tumor stage.^37^, ^38^, ^39^, ^40^ In melanoma, decreased PRDX2 expression promotes tumor growth and is linked to epithelial–mesenchymal transition and β-catenin signaling activation.^40^ Additionally, PRDX2 inhibition has been shown to enhance cisplatin sensitivity in AGS and SNU-1 cells, highlighting its potential as a therapeutic target in GC.^41^ Meanwhile, DDX27, a critical factor in ribosomal RNA processing, has been implicated in carcinogenesis across multiple cancers, including colorectal cancer, hepatocellular cancer, and GC.^42^^,^^43^ The prognostic significance of PRDX2 and DDX27 was further validated in an independent cohort of 75 GSRCC cases, reinforcing their potential as biomarkers for personalized disease management.

This study also examined a cohort of 79 LMT-GSRCC patients, revealing three distinct clusters based on proteomic characteristics. Interestingly, the C-Mb cluster exhibited unique biological traits, including lower metabolic rates in tumor tissue, a deviation from the typical metabolic characteristics of GSRCC. This finding aligns with emerging evidence suggesting that cancer cells prioritize metabolic efficiency, conserving energy for proliferation while minimizing extraneous cellular processess.^44^ Such adaptability may facilitate tumor metastasis by allowing cells to thrive in diverse microenvironments. However, the relatively small sample size for each subtype warrants further validation.

In an effort to identify potential therapeutic targets for underserved patient cohorts, we conducted a targeted protein screen for cluster 2, which demonstrated the worst prognosis. This analysis identified four FDA-approved drug targets, presenting new opportunities for GSRCC treatment. Among them, PFAS emerged as a particularly promising target in LMT-GSRCC. To explore its therapeutic potential, we performed virtual screening, identifying several candidate drugs, including temsirolimus, bosutinib, infigratinib, pralsetinib, and neratinib. Notably, neratinib exhibited strong therapeutic potential. PFAS, a key component of the “Purinosome” protein complex, plays a crucial role in *de novo* purine biosynthesis.^45^ Activation of the mitogen-activated protein kinase (MAPK)/extracellular signal-regulated kinase (ERK) pathway stimulates PFAS phosphorylation at Thr2 via ERK2, facilitating the production of purine intermediates essential for cell proliferation and tumor growth. Our findings suggest that neratinib-mediated inhibition of PFAS may offer a novel therapeutic strategy for LMT-GSRCC. Future studies should integrate pathway enrichment analyses of LMT-GSRCC subtypes for further mechanistic insights.

In summary, this study utilized global proteomic profiling of standard FFPE specimens, integrated with clinical outcome data, to characterize the heterogeneity of GSRCC. By correlating proteomic subtyping with clinical prognosis, we identified four distinct GSRCC subtypes. Furthermore, this is the first study to specifically focus on the LMT-GSRCC population, uncovering potential biomarkers and drug targets through proteomic analysis. These findings provide a foundation for developing novel targeted therapies and personalized treatment strategies for GC.

## CRediT authorship contribution statement

**Zhiyuan Jin:** Writing – review & editing, Writing – original draft, Methodology, Formal analysis. **Li Yuan:** Visualization, Software, Data curation. **Yubo Ma:** Data curation. **Zu Ye:** Resources. **Zhao Zhang:** Visualization, Software. **Yi Wang:** Methodology. **Can Hu:** Methodology. **Jinyun Dong:** Software. **Xinuo Zhang:** Resources. **Zhiyuan Xu:** Visualization. **Yian Du:** Resources. **Xiaoqing Guan:** Visualization. **Guangzhao Pan:** Validation. **Sichao Tian:** Writing – original draft. **Juan Li:** Funding acquisition, Formal analysis. **Ruiwen Zhang:** Writing – review & editing, Conceptualization. **Jiangjiang Qin:** Writing – review & editing, Project administration, Conceptualization. **Xiangdong Cheng:** Supervision, Project administration, Funding acquisition.

## Ethics declaration

This study was approved by the Research Ethics Committees of Zhejiang Cancer Hospital (Approval No. IRB-2022-371). The animal experiments were reviewed and approved by the Ethics Committee of the Hangzhou Institute of Medicine, Chinese Academy of Sciences (Approval No. AP2024-09-0199), and an ethical clearance certificate was obtained. All authors reviewed the manuscript and approved its submission.

## Data availability

Raw proteomic reads have been deposited in the China National Center for Bioinformation (Accession Number: Bioproject PRJCA017440). Data supporting the results of this study are available from the corresponding authors upon reasonable request.

## Funding

This work was supported by the 10.13039/501100012166National Key Research and Development Program of China (No. 2021YFA0910100, 2021YFA0910101), the Zhejiang Provincial Research Center for Upper Gastrointestinal Tract Cancer (China) (No. JBZX-202006), the Innovation Team and Talents Cultivation Program of the National Administration of Traditional Chinese Medicine (China) (No. ZYYCXTD-C-202208), the Medical Science and Technology Project of Zhejiang Province, China (No. WKJ-ZJ-2202, WKJ-ZJ-2104), the National Natural Science Foundation of China (No. 82074245, 81973634, 82204828), the Natural Science Foundation of Zhejiang Province, China (No. QKHM25H3103, LHDMY22H160008), the Zhejiang Leading Innovation and Entrepreneurship Team (China) (No. 2022R01006), the Pioneer R&D Program of Zhejiang, China (No. 2024SDYXS0003, 2023SDYXS0001), and the Program of Zhejiang Provincial TCM Sci-Tech Plan (China) (No. GZY-ZJ-KJ-24064).

## Conflict of interests

Dr. Yi Wang and Ruiwen Zhang are editorial board members for *Genes & Diseases* and were not involved in the editorial review or the decision to publish this article. Other authors declare that there are no competing interests.
