# Supplementary material for: A comprehensive proteomic analysis uncovers novel molecular subtypes of gastric signet ring cell carcinoma: Identification of potential prognostic biomarkers and therapeutic targets
Source: Genes Dis. 2025 Jun 14;13(1):101717. doi: 10.1016/j.gendis.2025.101717 (PMC12557601; doi:10.1016/j.gendis.2025.101717)
Supplement: Multimedia component 1 [file mmc1.docx]

*Supplementary Materials*

**A Comprehensive Proteomic Analysis Uncovers Novel Molecular Subtypes of Gastric Signet Ring Cell Carcinoma: Identification of Potential Prognostic Biomarkers and Therapeutic Targets**

Zhiyuan Jin^a,b,1^, Li Yuan^b,c,d,1^, Yubo Ma^b^, Zu Ye^b^, Zhao Zhang^e^, Yi Wang^b^, Can Hu^b^, Jinyun Dong^b^, Xinuo Zhang^b^, Zhiyuan Xu^b,c,d^, Yi-An Du^b,c,d^, Xiaoqing Guan^b^, Guangzhao Pan^b^, Sichao Tian^f^, Juan Li^b^, Ruiwen Zhang^g^, Jiang-Jiang Qin^b,c,d,**^, Xiangdong Cheng^b,c,d,*^

**^*^ Corresponding Author**: Xiang-Dong Cheng, Department of Gastric Surgery, Zhejiang Cancer Hospital, Banshan Road 1#, Hangzhou, Zhejiang 310022, China; Email: [chengxd@zjcc.org.cn](mailto:chengxd@zjcc.org.cn); Tel/Fax: +86-571-87070965.

**^**^ Corresponding Author**: Jiang-Jiang Qin, Hangzhou Institute of Medicine (HIM), Chinese Academy of Sciences, 150 Dongfang Road, Hangzhou, Zhejiang 310018, China; Email: [jqin@ucas.ac.cn](mailto:jqin@ucas.ac.cn); Tel/Fax: +86-571-88121791.

**Supplementary Tables**

**Table S1.** Clinicopathological information of the patients.

**Table S2.** The propensity score matching was performed at a ratio of 1:1.

**Table S3.** The propensity score matching was performed at a ratio of 1:4.

**Table S4.** Clinicopathological information of 112 patients with GSRCC.

**Table S5.** Clinicopathological information of 79 patients with LMT-GSRCC.

**Table S6.** PCT-assisted protein extraction results for each sample.

**Table S7.1.** Protein intensity data for 112 GSRCC patient samples.

**Table S7.2.** Log-transformed protein intensity data for 112 GSRCC patient samples.

**Table S8.1.** Input for differential protein analysis between cancer and adjacent tissues (Paired two-sided Welch's t-test).

**Table S8.2.** Downregulated proteins in tumor vs. adjacent tissues (Fc > 1.5 & p < 0.05).

**Table S8.3.** Upregulated proteins in tumor vs. adjacent tissues (Fc > 1.5 & p < 0.05).

**Table S9.** Differentially expressed proteins across TNM stagess (I, II, Ⅲ, IV).

**Table S10.** Clustering of differentially expressed proteins across TNM stages (I, II, III, IV) using the mfuzz algorithm.

**Table S11.1.** Downregulated proteins in the LP vs. SP group (Fc > 1.2 & p < 0.05).

**Table S11.2.** Upregulated proteins in the LP vs. SP group (Fc > 1.2 & p < 0.05).

**Table S12.** Sample typing results processed using the NMF algorithm.

**Table S13.** Protein ratio and p-values for subtypes vs other subtypes comparisons (N = 112).

**Table S14.** "Tumor vs. adjacent tissue" ratio values for the top 100 most differentially expressed proteins across subtypes (N = 112).

**Table S15.** Top 100 most significantly differentially expressed proteins in each subtype (N = 112).

**Table S16.** Expression levels of all proteins after complementation.

**Table S17.** Detailed clinical information of the validation cohort.

**Table S18.** Expression levels and prognostic significance of PRDX2 and DDX27 in an independent validation cohort of 75 GSRCC cases (N = 75)

**Table S19.** Sample typing results processed using the NMF algorithm through a non-negative matrix.

**Table S20.** Protein ratio and p values for subtype vs. other subtype comparisons (N = 79).

**Table S21.** "Tumor vs. adjacent tissue" ratio values for the top 100 most differentially expressed proteins across subtypes (N = 79).

**Table S22.** Top 100 most significantly differentially expressed proteins in each subtype (N = 79).

**Table S23.** Significantly upregulated proteins of three subtypes.

**Table S24.** Significantly upregulated proteins in subtype 2 of LMT-GSRCC (ratio>1.5, p<0.05).

**Table S25.** Molecular docking results of protein-small molecule interactions using MOE, with drug candidates ranked by score.

Table S26. Multivariate Cox proportional hazards regression of Cluster 2-specific signature proteins.

**Supplementary Figures and Figure Legends**


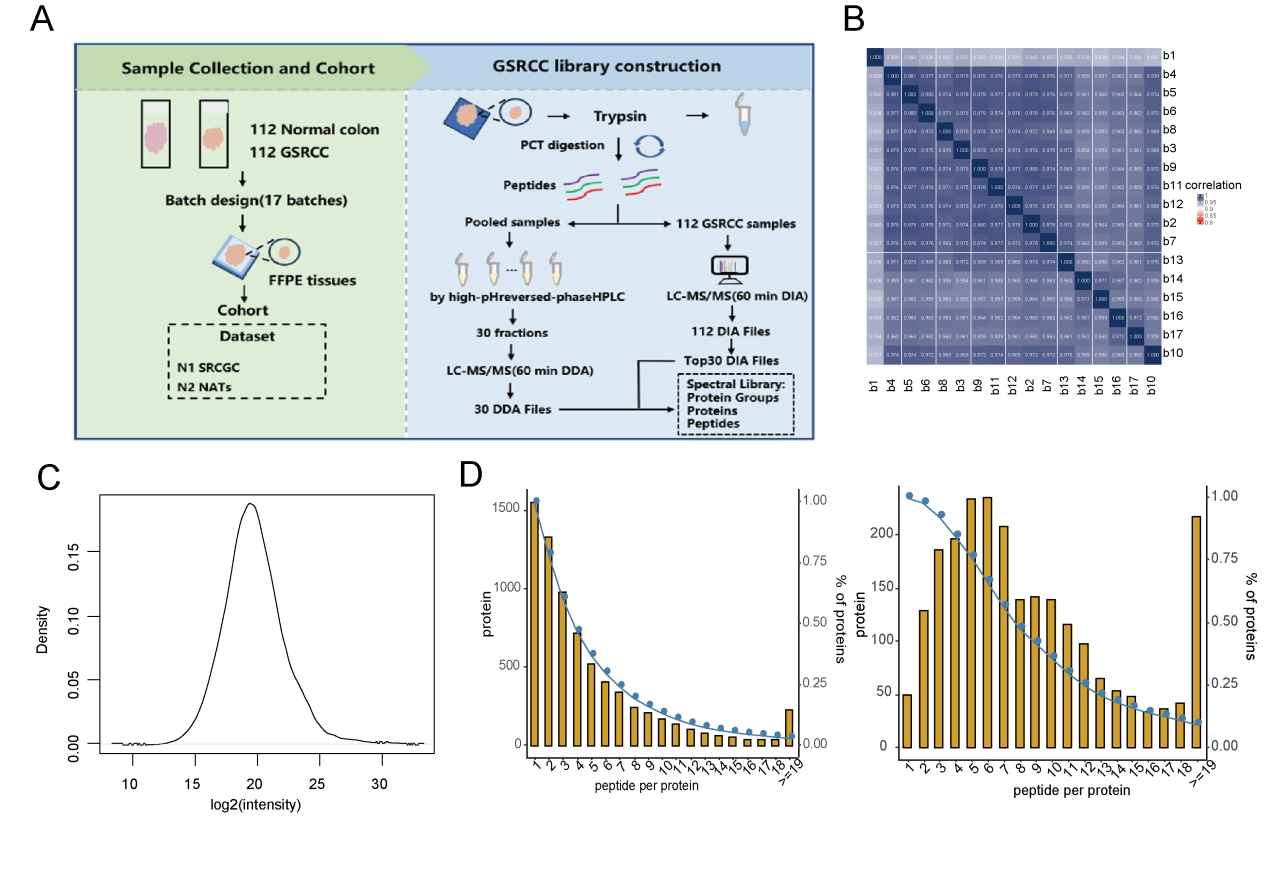


**Fig. S1.** Sample pretreatment and quality control for gastric signet ring cell carcinoma patients. **(A)** Schematic diagram of the proteomics method design. **(B)** Heatmap of the correlation coefficient matrix. The number in each circle represents the Pearson correlation coefficient between the two corresponding variables. The magnitude of the correlation coefficient corresponds to the color intensity of the color bar. All pooled samples had correlation coefficients greater than 0.9. **(C)** Original data density distribution. The abscissa represents the relative expression abundance of proteins, while the ordinate data indicates the relative density of the data. No significant outliers were observed in the original data from 112 paired samples. **(D)** The overall profile of proteins identified through different peptide alignments. The left panel shows all identified proteins, while the right panel shows proteins that could be identified in all samples. Blue dots represent the percentage of total proteins identified by the minimum number of peptides. The X-axis shows the number of peptides corresponding to each protein, the left Y-axis represents the number of proteins, and the right Y-axis shows the percentage of proteins identified by the least number of peptides relative to the total number of proteins.


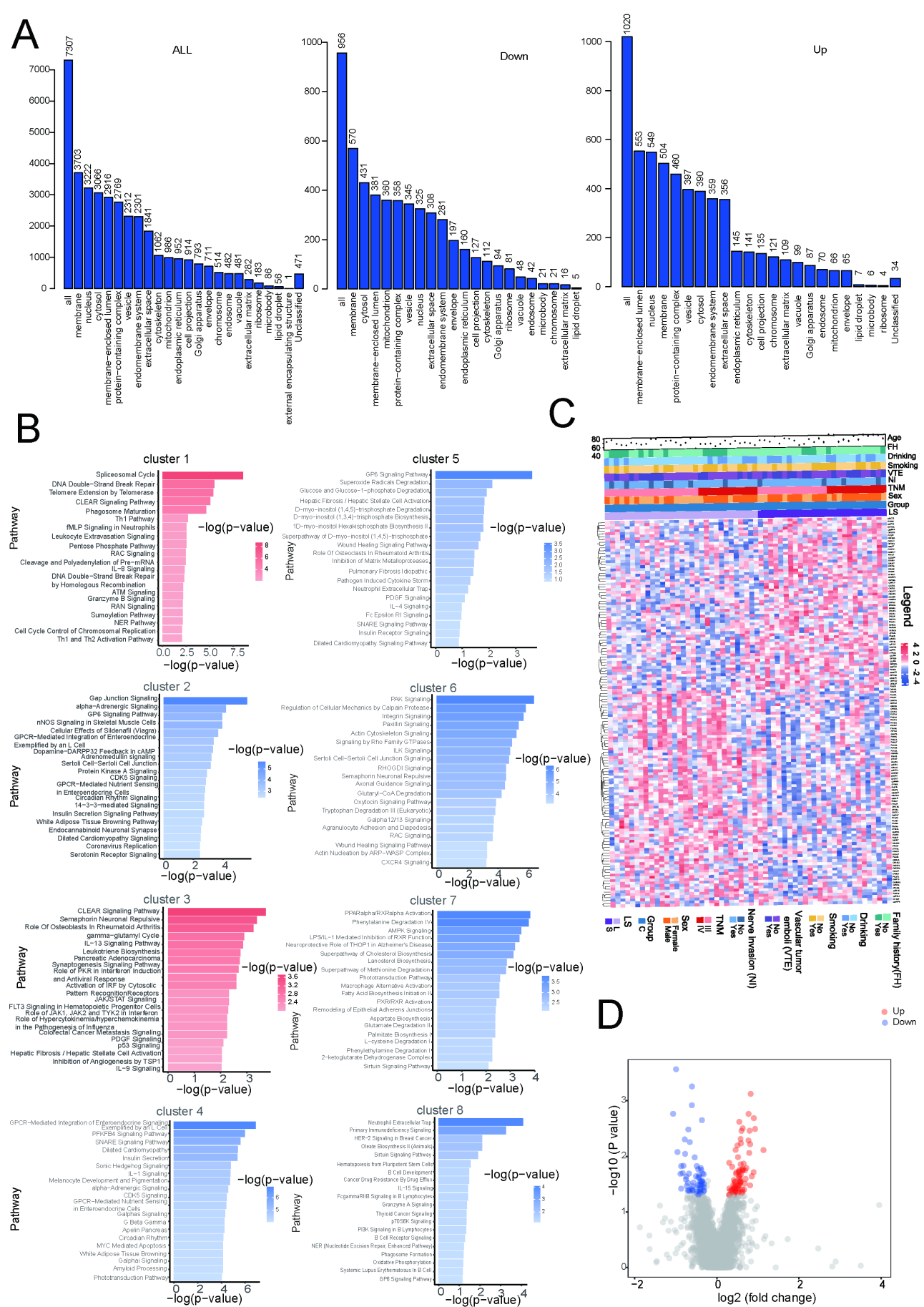


**Fig. S2.** Stratification and analysis of GSRCC proteomic data. **(A)** GO analysis revealed the pathways significantly enriched in different proteomic subtypes. **(B)** IPA was used to enrich the pathways associated with differentially expressed clusters. **(C)** Heatmap analysis comparing long survival and short survival groups. **(D)** A volcano plot was used to display differentially expressed proteins associated with long or short prognosis. The horizontal axis represents Log2 (fold change), and the vertical axis represents -Log 10 (adjusted P-value). Red indicates upregulated proteins, green indicates downregulated proteins, and gray indicates proteins with no significant change.


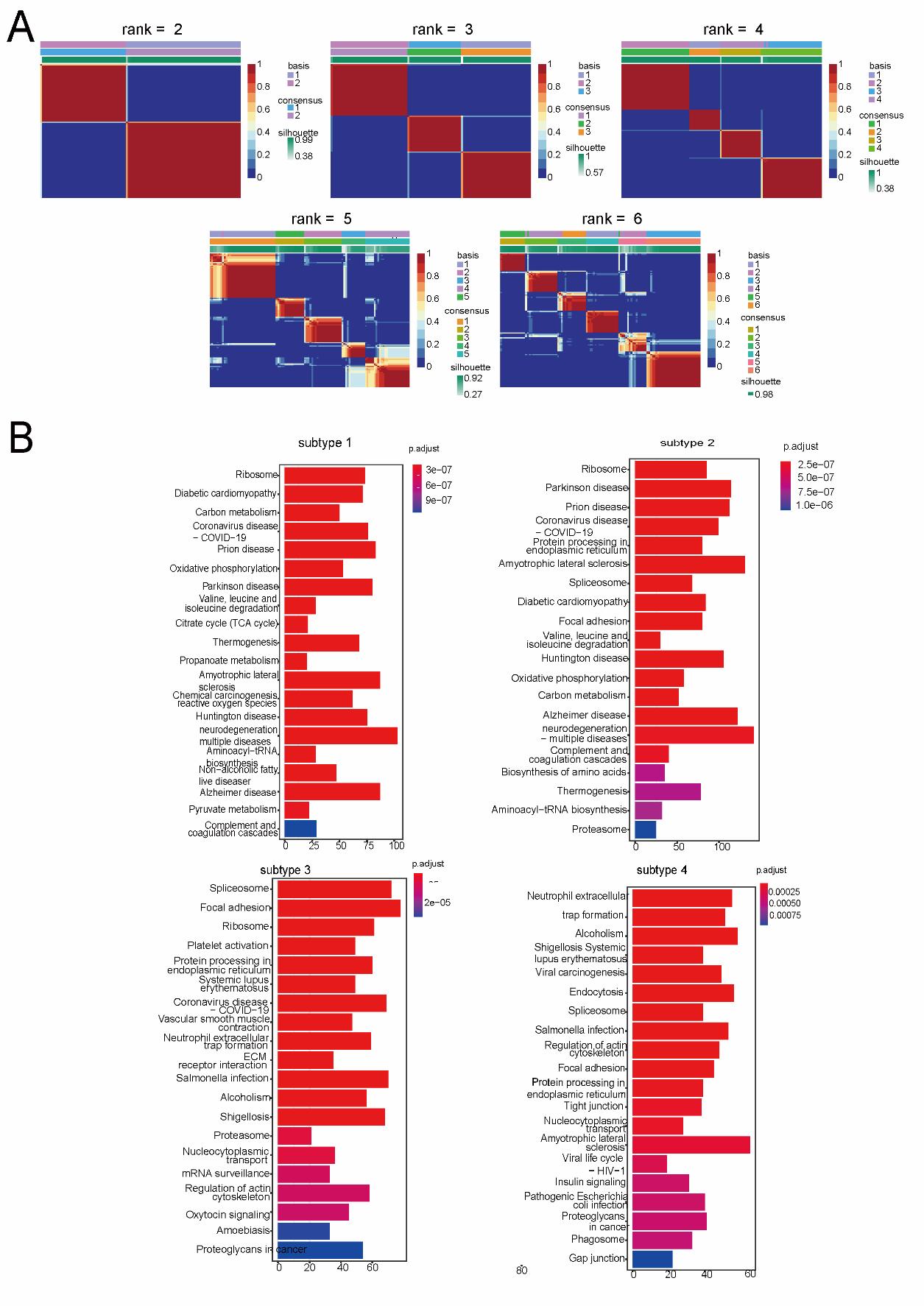


**Fig. S3.** Unsupervised proteomic analysis of GSRCC identifies distinct clusters. **(A)** Non-negative matrix factorization (NMF) of 112 cases extracted proteins based on the “Tumor to NATs” ratio. **(B)** Gene set enrichment analysis (GSEA) using the KEGG subset of canonical pathways revealed significantly enriched pathways across different proteomic subtypes.


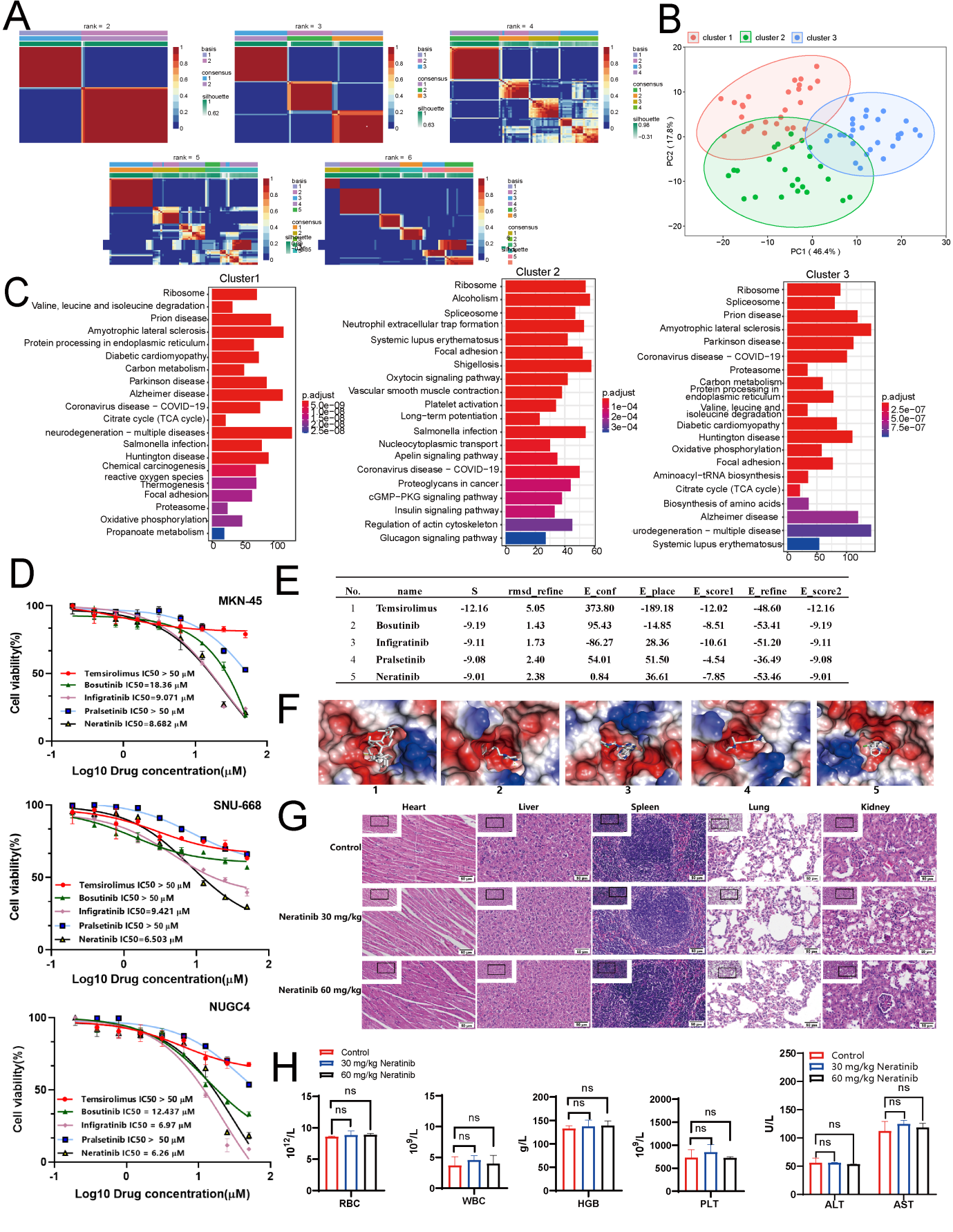
**Fig. S4.** Unsupervised proteomic analysis of LMT-GSRCC identifies distinct clusters. **(A)** NMF of 79 cases extracted proteins based on “Tumor to NATs” ratio. **(B)** PCA plots depicting different clusters. **(C)** GSEA identified pathways significantly enriched in different proteomic clusters. **(D)** Cytotoxicity assays of five drugs at varying concentrations in gastric signet ring cell lines. **(E)** Molecular docking model illustrating drug binding to PFAS domains, with docking scores for the five selected drugs. **(F)** Surface presentation of drug binding to the PFAS protein domain, with key residues shown as rods and hydrogen bonds indicated by yellow dashed lines. **(G)** Histopathological examination of the heart, liver, spleen, lungs, and kidneys using HE staining to evaluate the potential toxicity of Neratinib. **(H)** Peripheral venous blood was collected from tumor-bearing mice treated with or without Neratinib for complete blood count analysis, comparing white blood cell (WBC) count, red blood cell (RBC) count, hemoglobin (HGB) levels, and platelet (PLT) count between the two groups. Serum was isolated to assess alanine transaminase (ALT) and aspartate transaminase (AST) levels.
